# A fully autonomous terrestrial bat-like acoustic robot

**DOI:** 10.1371/journal.pcbi.1006406

**Published:** 2018-09-06

**Authors:** Itamar Eliakim, Zahi Cohen, Gabor Kosa, Yossi Yovel

**Affiliations:** 1 Mechanical Engineering Department, Tel Aviv University, Tel Aviv, Israel; 2 Intelligent Medical Micro/Nano Systems Group, University Hospital of Basel, Basel, Switzerland; 3 Sagol School of Neuroscience, Tel Aviv University, Tel Aviv, Israel; 4 School of Zoology, Faculty of Life Sciences, Tel Aviv University, Tel Aviv, Israel; Northeastern University, UNITED STATES

## Abstract

Echolocating bats rely on active sound emission (echolocation) for mapping novel environments and navigating through them. Many theoretical frameworks have been suggested to explain how they do so, but few attempts have been made to build an actual robot that mimics their abilities. Here, we present the ‘Robat’—a fully autonomous bat-like terrestrial robot that relies on echolocation to move through a novel environment while mapping it solely based on sound. Using the echoes reflected from the environment, the Robat delineates the borders of objects it encounters, and classifies them using an artificial neural-network, thus creating a rich map of its environment. Unlike most previous attempts to apply sonar in robotics, we focus on a biological bat-like approach, which relies on a single emitter and two ears, and we apply a biological plausible signal processing approach to extract information about objects’ position and identity.

## Author summary

Many animals are able of mapping a new environment even while moving through it for the first time. Bats can do this by emitting sound and extracting information from the echoes reflected from objects in their surroundings. In this study, we mimicked this ability by developing a robot that emits sound like a bat and analyzes the returning echoes to generate a map of space. Our Robat had an ultrasonic speaker mimicking the bat’s mouth and two ultrasonic microphones mimicking its ears. It moved autonomously through novel out-doors environments and mapped them using sound only. It was able to negotiate obstacles and move around them, to avoid dead-ends and even to recognize if the object in front of it is a plant or not. We show the great potential of using sound for future robotic applications.

## Introduction

The growing use of autonomous robots emphasizes the need for new sensory approaches to facilitate tasks such as obstacle avoidance, object recognition and path planning. One of the most challenging tasks, faced by many robots, is the problem of generating a map of an unknown environment, while simultaneously navigating through this environment for the first time [1]. This problem, is routinely solved by echolocating bats that perceive their surroundings acoustically (other animals also solve this task on a daily basis using a range of sensory modalities) [2]. By emitting sound signals and analyzing the returning echoes, bats can orient through a new environment and probably also map it [3] [4]. Inspired by this ability, we present the ‘Robat’—a fully autonomous terrestrial robot that solely relies on bat-like SONAR to orient through a novel environment and map it. Using a biologically plausible system with two receivers (ears) and a single emitter(mouth) which produced frequency modulated (FM) chirps at a typical bat rate, the Robat managed to move through a large out-doors novel environment and map it in real-time.

There have been many attempts to use airborne sonar for mapping the environment and moving through it using non-biological approaches; for example by using an array of multiple narrow-band speakers [5, 6] [7] and or multiple microphones [8]. These studies proved, that by using multiple emitters, or by carefully scanning the environment with a sonar beam, as if it were a laser, one can map the environment acoustically, but these approaches are very far from the biological solution [9]. A bat emits relatively few sonar emissions towards an object, and it must rely on two receivers only (its ears) in order to extract spatial information from its very wide bio-sonar beam which can reach 60 degrees (6 dB double side drop in amplitude [10] [11] [12]). Unlike the narrow-band signals typically used in robotic applications, the bat’s wide-band signals provide ample spatial information allowing it to localize multiple reflectors within a single beam. This is the approach we aimed to test and mimic in this study.

Numerous studies have shown that echoes generated by emitting bat-like sonar signals contain spatial information that can be exploited for localization and identification of objects [13] [14] [15] [16] [17] [18]. Several previous attempts have been made to model and mimic bats’ spatial abilities of localization and mapping [19] [20]. One of the most comprehensive attempts to use a biological approach to map the environment was ‘BatSLAM’ [21], which relied on mammalian brain-like computation for simultaneous localization and mapping of a novel environment using biomimetic sonar. Using a biological representation of the data (the cochleogram) the BatSLAM algorithm generated topological maps in which the nodes represent unique places in the environment and the edges represent the robot’s displacements between them. The approach of recognizing a location based on its unique acoustic signature was further broadened by Vanderelst et al. [6] who classified a wide range of natural scenes based on their acoustic statistics, once again, without extraction of their spatial characteristics. Vanderelst et al. limited the information extracted from the echoes to the acoustic resolution available to a bat, and they were still successful in achieving useful scene recognition.

Our work differs from these former studies in two important respects: (1) Our Robat moved through the environment autonomously while the previous robots were driven by the user. (2) We mapped the 2D structure of the environment, while they mapped the position of the robot in the environment. Namely, in our approach the outline of the objects that were encountered by the Robat were delineated so that paths (free of obstacles) were revealed for future use. In these previous studies, objects in the environment were mapped as locations with a unique acoustic representation so that when encountered again, the agent could localize itself on the acoustic-map, but no spatial information about objects’ size or orientation was extracted. When moving autonomously, such information is essential for movement planning.

In addition to mapping, our Robat had to autonomously move through the environment while avoiding obstacles. Some previous attempts were made to model orientation and obstacle avoidance using a biological echolocation-based approach. For example, Vanderelst et al. [9], suggested a simple sensorimotor approach for obstacle avoidance based on turning away from the louder of the two echoes received by the ears. They showed that a simulated agent can move through a novel environment without any mapping of the positions or borders of the objects within it. This approach might be beneficial when an animal wants to move fast through the environment without an intention of returning to specific locations within it, but if the animal needs to find its way back to some point in this environment (e.g., to its roost), or to plan its movement to a specific location, some mapping must be performed. For example, the robust low-level sensorimotor heuristic presented in [9] could be combined with higher level mapping algorithms (e.g., [22]).

To our best knowledge, our Robat is the first fully autonomous bat-like biologically plausible robot that moves through a novel environment while mapping it solely based on echo information—delineating the borders of objects and the free paths between them and recognizing their type.

## Results

**Fig 1:**
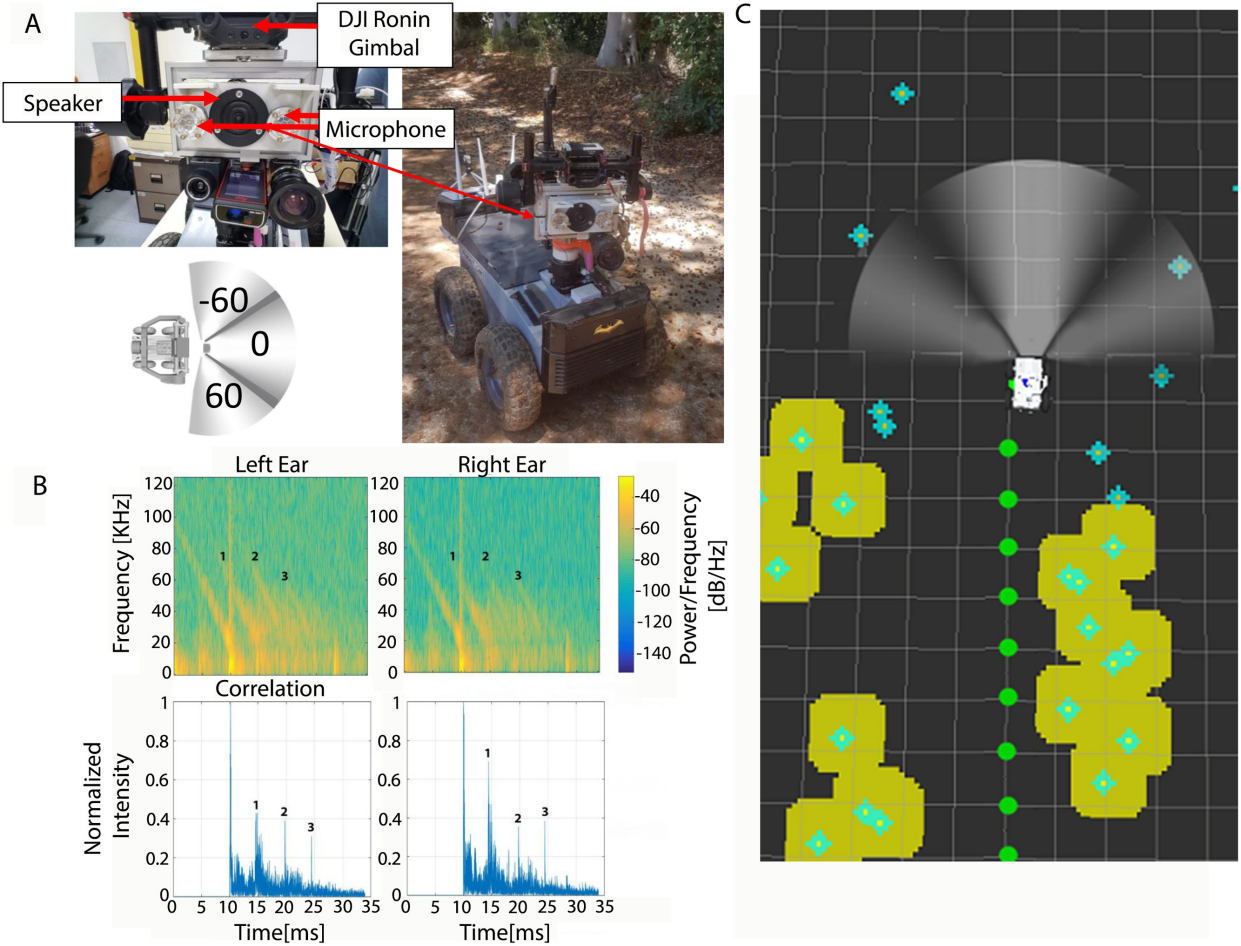
The system—The Robat, signal processing and mapping. (A) Image of the Robat. Insert shows the Robat’s sensory unit including a speaker and two receivers. (B) An example of a single echo received by the Robat’s two ears. Top row shows right and left ear echo spectrograms where the emitted signal and 3 consecutive echoes can be observed. The first and loudest peak is the emitted signal. Bottom row shows the correlation signals with the peaks that were detected as returning from the same object numbered in each ear (1-3). (C) The Robat’s acoustic mapping. Objects recognized by the Robat are shown as turquoise points. Yellow shading shows the inflation of objects into a map deliniating the borders of the route. Note that turquoise points ahead of the robot have not yet been inflated, because inflation does not occur at each acquisition. Green dots show the locations of echo acquisition points (every 0.5m). The three directions of acquisition are depicted by the three-parts beam.

The Robat’s goal was to move through an environment that it has never experienced before, finding its path between vegetation and other obstacles while mapping their locations, delineating their borders and identifying them (when possible) similar to a bat flying through a grove or a shrubbery which it encounters for the first time (Fig 1A).

### Acquisition

The Robat moved through the environment emitting echolocation signals every 0.5m thus mimicking a bat flying at 5m/s while emitting a signal every 100ms which is within the range of flight-speeds and echolocation-rates used by many foraging bats [23] [24, 25] [25] [26]. Every 0.5m, the Robat emitted three bat-like wide-band frequency-modulated sound signals while pointing its sensors (emitter and receivers) in three different headings: -60, 0, 60 degrees relative to the direction of movement (Fig 1A). This procedure aimed to overcome the narrow acoustic beam of the Robat and to better mimic a bat beam which is typically much wider than that of our speaker (see Methods) [27] [28] [10] [29] [11] [12].

### Mapping

Following echo acquisition, acoustic peaks of interest (representing objects) were identified in the echoes (Fig 1B). Equivalent peaks—i.e., peaks returning from the same object—received by the two ears were matched and the reflecting objects were localized. The time-delay between the emission and the arrival of the echoes was used to determine the distance of an object and the difference between the time of arrival of the echo to the two ears was used to determine its azimuth (i.e., Mapping was performed in 2D, Fig 1C, Turquoise points depict objects’ location, see Methods for full details). Importantly, the Robat was able to localized multiple objects whose echoes were received within a single beam (S1 Fig). This ability has not been reported in previous studies and bats are likely able to do so. After every 5 steps (i.e., 2.5m) the Robat applied an inflation and interpolation algorithm that incorporated the newly mapped objects into the map that has been created so far (based on the previous echoes, Fig 1C, yellow shaded area, see Methods). At each time step, following echo acquisition and object localization, the Robat planned its next movement according to the iterative map that has been created so far and according to the objects detected in the most recent acquisition. Movement planning was based on the bug algorithm [30] which can be simply described as turning 90 degrees to the right, whenever an obstacle is encountered ahead, and then turning left to maneuver around the obstacle.

The movement and mapping algorithms were tested in two outdoor environments: (1) The pteridophyte greenhouse (5m x 12m) and (2) The palm greenhouse (40m x 5m) both situated in the Tel Aviv University Botanical Garden.

**Fig 2:**
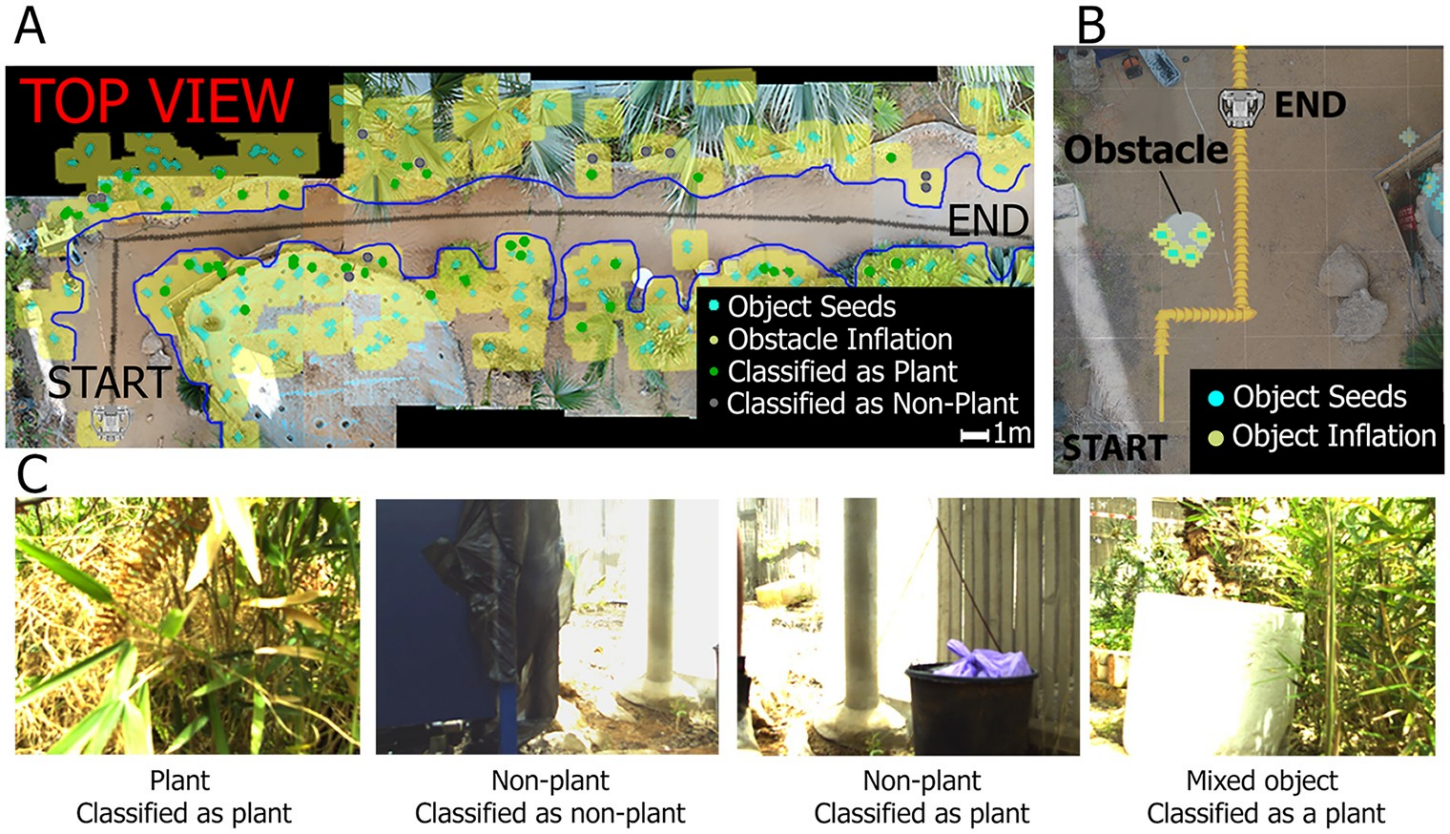
Mapping and obstacle avoidance. (a) Robat’s mapping of the greenhouse overlaid on drone images. Black line depicts the trajectory taken by the Robat. Yellow shaded areas show the objects mapped by the Robat and the blue line shows their borders. Turquoise points depict the center of the objects as they have been localized by the Robat. (b) Passing a cylinder obstacle (D = 0.8m) that has been placed in the Roba’ts way. Yellow arrows show the trajectory taken by the Robat to pass the obstacle (doing so fully autonomously). Turquoise points depict points on the obstacle as they have been localized by the Robat. (c) Examples of object classification. Two correct classifications and two wrong ones are presented. Note that the ‘non-plant’ classified as a plant includes a bamboo mesh. Such mesh objects create echoes that have plant-like acoustic characteristics.

The Robat successfully moved through both new environments without hitting objects and while mapping their locations and contour line (see Robat’s trajectory depicted in black in Fig 2A). When an obstacle was placed in the Robat’s way, it moved around it (Fig 2B). To quantify the mapping performance, we compared the contour of the objects as it was estimated by the Robat to the real contour (which we estimated from drone images in the Palm greenhouse and measured manually in the Pteridophyte greenhouse, see Methods). In the palm greenhouse, the mean distance between the two contours was 0.42 ± 0.74 (mean + STD) [m] meaning that along the 35m trail that the Robat passed and mapped in the Palm greenhouse, the estimated borders of the objects on both sides of the trail, were off by 42cm on average, relative to their real position. This might seem inaccurate when considering bats’ ability to estimate range with an accuracy of less than 1cm in a highly controlled experiment, [31] [32] but it should be emphasized that the Robat only detected and localized parts of the objects while their borders were delineated based on our inflation an interpolation algorithm (Methods). Moreover, note that many of the objects in our environment were plants with multiple branches so that the exact borders of the objects were inherently difficult to define (even in the drone images). Similar performance (0.44 ± 0.25 (mean + STD) [m]) was observed in the second environment (the Pteridophyte greenhouse, S2 Fig).

### Classification and decision making

When moving through the environment, a real bat can probably use echoes in order to classify objects into categories (e.g., rocks, trees, bushes) and even to identify specific objects (e.g., a specific beech tree in its favorite foraging site). Such recognition would greatly assist the bat to navigate, for example, by recognizing specific landmarks at important turning points along its flight route and it could also assist its foraging, for example, by recognizing specific vegetation that is rich in fruit or insects [33] [17]. So far we demonstrated that the Robat can translate a novel natural environment into a binary map of open spaces and obstacles. In order to improve the mapping, we added a classification step to the algorithm, which was performed using a neural-network that was trained to distinguish between two object categories—plants and non-plants. To this end, a set of acoustic features were extracted from the echoes and used as input for the network (Methods). The Robat was able to classify objects as plants or non plants significantly above chance level (Fig 2C and Table 1) with a balanced accuracy of 68% (chance was 50%, P = 0.01, based on a permutation test with 100 permutations, the balanced accuracy is the number of correct classifications in each class, divided by the number of examples in each class, averaged over all classes. This measurement mitigates biases which could rise from unbalanced class sizes, see Methods). The classification performance is also shown in Fig 2a where colored points depict plants (green) and non-plants (gray).

**Table 1:** Classification performance on the test set, for the plant vs. non-plant task.

True Label / Predicted Label	Plant	Non-Plant
Plant	77%	23%
Non-Plant	42%	58%

Finally, we tested the functionality of this classification ability by purposefully driving the Robat into a dead end where it faced obstacles in all directions ahead (i.e., right, left and straight ahead, S7 Fig). The Robat had to determine which of the three obstacles was a plant, through which it could drive, and it did so successfully at ~70% of the cases (in accordance with its ~70% accurate classification rate, see movie: https://www.youtube.com/watch?v=LzGGuzvYSH8-second 49 and onward).

## Discussion

In this study, we managed to build an autonomous robot that moves through a novel environment and maps it acoustically using bat-like Bio-sonar. We achieved high mapping accuracy, despite our simple approach, proving the great potential of using active wide-band sound emissions to map the environment. We created a (2D) topographic map which would allow us to plan future movements through the environment (and not a topological map). The statistical approach presented in [9] is therefore complementary to ours, allowing classifying specific locations based on their echoes. For example, when navigating back to a specific location using the map created by the Robat, their approach could be used to validate the arrival at the desired location and also to help adjust the map to improve its accuracy.

The Robat was much slower than a real bat, stopping for ca. 30 seconds every 0.5m to acquire echoes. This slowness was however, merely a result of the mechanical limitations of our system and mainly the gimbal that was slow. Using a speaker with a wider beam (that eliminates the need to turn at each location) would allow the Robat to acquire echoes on the move, while moving as fast as a bat. Importantly, despite our stopping for echo recording, the acoustic information we acquired did not differ from that received by a bat, except for the fact that a bat’s echoes would also be slightly Doppler-shifted (but this would probably not affect any of our results).

In some respects, our processing was not fully bat-like. We used a sampling rate of 250kHz, which is higher than the theoretical time precision of the auditory system [34]. Bats and other small mammals have been shown to estimate azimuth with an accuracy of <10degrees (the exact accuracy depends on the azimuth, (e.g. [35, 36])). This accuracy accounts for an inter-aural time difference of <10*μ*s which is in accordance with our sampling rate (sampling at 250kHz is equivalent to an error of ~5*μ*s when estimating time differences between two ears). Therefore, even if our computation was different from that of a bat (which does not cross-correlate two highly sampled time signals) the overall accuracy allowed by our approach was not better than that of a bat. Moreover, due to the inflation and interpolation method that we used in order to delineate the borders of the objects, the effective accuracy of our mapping was much lower than that allowed by this high sampling rate, and probably much lower than that available to bats [31, 32]. Therefore, we hypothesize that using an auditory preprocessing model like that used in Batslam for example [21] would probably not change our results dramatically. Another advantage that we had over real bats was the relatively large distance between the two ears which were spaced 7cm apart—ca. two times more than in a large bat. This probably allowed more accurate azimuth estimations, but once again, we hypothesize that because of the use of inflation, this did not improve our performance dramatically. Importantly, we managed to extract information about multiple objects within a single sonar beam. On average, in each echo that contained reflections (some echoes did not) we detected 4.1 objects positioned in a range of azimuths between -50—50 degrees. Another important difference between the Robat and an actual bat is the lack of an external ear in the Robat. The angle-dependent frequency response of the external ear allows bats (and other animals) to gain information about the location of a sound source in three dimensions. Because we relied on temporal information for object localization, we used a first approximation of an ear. Adding a structure mimicking the external ear could have further improved our localization performance and it would be essential in order to expand our mapping to 3D.

In order to better mimic the bat’s beam, we used three beams (directed 60 degrees apart), but this made our task easier than a bat’s because we could analyze the echoes returning from each direction separately. We therefore also tested an approach in which we sum the three echoes collected (with different headings) at each acquisition point, thus mimicking a wider beam. Even with this degraded data, we were able to map the environment with a decent accuracy of 1.14 ± 0.70 [m] (mean + STD, S6b Fig), an accuracy that would allow future planning of trajectories while avoiding obstacles on the way.

In some respects our approach was probably much more simplistic than a bat. For example, the obstacle avoidance algorithm was very simple and a better approach would probably use control-theory to steer the Robat around obstacles [37]. In terms of mission priority, we used serial processing where the Robat first processes new incoming sensory information; it then performs the urgent low-level task of obstacle avoidance and path planning, and only every several acquisitions, it performs the high-level process of map integration. There is much evidence that the mammalian brain also performs sensory tasks sequentially (e.g., [38]) but it would be interesting to test some procedures for parallel processing in the future.

In addition to mapping the positions of objects in the environment, a complete map should also include information about the objects such as their type or identity. To show that such information is available in the echoes, we developed a classifier that can categorize objects based on their echo. We hypothesize that the medium classification performance that we achieved (68%) was a result of our choice of categories. We trained the classifier to distinguish between plant and non-plant objects but these are not always two well distinct groups. For example, the echo of an artificial object such as a fence will have vegetation-like acoustic features and indeed most of the classifier’s mistakes were recognition of non-plants as plants. Bats might thus divide their world of objects differently, perhaps to diffusive vs. glint-reflecting objects.

Altogether, we show how a rather simple signal processing approach allows to autonomously move and map a new environment based on acoustic information. Our work thus proves the great potential of using acoustic echoes to map and navigate, a potential that is translated into action by echolocating bats on a daily basis.

## Materials and methods

### Acquisition

The Robat was based on the ‘Komodo’ robotic platform (Robotican, Israel). The Bio-sonar sensor was mounted on a DJI Ronin gimbal which allowed turning the sensing unit relatively to the base of the robot in a stable manner. The sensing unit included an ultrasonic speaker acting as the bat’s mouth (VIFA XT25SC90-04) and 2 ultrasonic microphones acting as the bat’s ears spaced 7cm apart (Avisoft-Bioacoustics CM16/CMPA40-5V Condenser). The speaker and the microphones were connected to A/D and D/A converters which were based on the USB-1608GX-2AO NI DAQ board, sampling at 250KS/s at each ear. The emitted signal was a 10ms FM chirp sweeping between 100-20kHz. It was amplified using a Sony Amplifier (XM-GS4). An uEye RGB camera, was used for image collection for validation purposes only. Three 2.4GHz/5.8GHz antennae were mounted at the rear of the Robat for wireless communication between the Robat and a stationary station. This allowed viewing the map created by the Robat in real time, but importantly, all calculations and decisions were performed on the Robat itself.

### Mapping

While moving, the Robat stopped every 0.5m (based on its odometry measurements) and the sonar system (emitter and receivers) was rotated to three different headings [0,60,-60 degrees] relative to the direction of movement, a sound signal (see above) was emitted, and echoes were recorded. Each recording was 0.035 sec long, equivalent to a range of ca. 6 meters (farther objects were thus ignored at each emission). The signal-to-echo delay time and the time of arrival differences of the echoes to the two ears (i.e., the Interaural Time Difference) were used together in order to map the environment. To this end, the received signals were cross-correlated with the theoretical emitted signal. The cross-correlated signal was normalized relative to the maximum value of the recording, and a peak detection function was used to find peaks of interest (python peakutil with a minimal peak distance of 0.002 sec, and a min amplitude of 0.3.).

To match peaks arriving at the right and left ears, for each peak detected in one ear, an equivalent peak was searched for in the other ear within a window of +/- 0.001 sec. If a peak was found, the Pearson correlation was used to determine if the two echoes were reflections of the same object. For this purpose, a segment of 0.01 seconds around each peak was cut and the correlation between the two time signals (one from each ear) was computed. Only correlations higher than 0.9 were accepted. This threshold was conservative thus potentially resulting in missing of objects, but it reduced the localization of artifact non-existent objects. Because the Robat emitted very 0.5 m—there was much overlap between echoes of consecutive emissions. We were therefore likely to detect an object several times, so a conservative approach was chosen. In addition to its position, each object on the map was defined by three parameters: “C |T |P”, where C is the Pearson correlation coefficient between the left and right ears for the specific point, T is the object’s type based on its acoustic classification—either artificial or a plant, and P is the classification probability (see more below about the classification process).

Results in the in-doors controlled environment showed that using two ears, the mean error in distance estimation was 1.3 ± 2.1 [cm] (mean + STD, S3 Fig) and the mean azimuth estimation error was 1.2 ± 0.7 [degrees] (mean+STD, S3 Fig). Importantly, these are the results for a single reflector, so accuracy in the real environment where many reflections are received at each point will be lower.

Every 5 Robat-steps, newly localized objects were integrated into the map that was created so far. This was done using an Iterative-Object-Inflation algorithm, which inflated points into squares and connected them. To this end, the entire area around the Robat was divided into a grid with 2000x2000 pixels (5x5cm^2^ each). Each detected object was placed in the corresponding pixel on the map and was inflated to an area of 20x20 pixels around its center (i.e., 1x1 m^2^, S4 Fig). This procedure creates a binary map with 1’s depicting objects and 0’s depicting a free path. Pixels along the trajectory that the Robat previously moved through always received the value 0 depicting an open path (even if they were within the 20x20 window of a detected object).

### Movement and obstacle avoidance

We chose a very simple obstacle avoidance approach also known as the ‘bug algorithm’ [39]. During the exploration process, the Robat moved forward in steps of 0.5m between consecutive acquisition points. When detecting an obstacle less than 1.2m in front of it, the Robat turned 90 degrees towards the right, and performed a 1m step towards the right (after checking that there is no obstacle ahead). After performing a 1m step to the right, the Robat turned 90 degrees to the left and acquired an echo. If no obstacle was detected (meaning that the obstacle has been passed) the Robat continued straight (i.e., in its previous direction before turning right). If the way was still blocked (i.e., the obstacle was not passed), the Robat turned again to the right and kept moving towards the right (90 degrees relative to its original direction).

### Summing echoes from all three headings

In order to better mimic the bat, that has a beam much wider than Robat’s beam, we examine an approach of summing the echoes returning from the three different headings (mentioned above) into one superposition echo, and then running the same (detection, localization and mapping) algorithms as described above.

### Evaluation of the mapping accuracy

In order to examine the acoustic map generated by the Robat, inspired by [40], we collected aerial images using a drone (DJI Phantom 4, DJI), to construct a complete ground truth map of the area. This procedure was only performed for the large palm greenhouse (40x5 m^2^). The contour of the objects on both sides of the trail in the greenhouse was extracted and compared to the contour of the inflated map that was acoustically reconstructed by the Robat (both contours were marked manually). Each of the two contours was fit by a 55-coefficient order polynomial function which was then sampled at 500 points to get a high resolution description of the contour. The two contours (real and Robat-estimated) were compared by calculating the root-mean-square distance between them (the average over these 500 points, S5 Fig).

### Classification

Acoustic based object classification was performed using a neural-network that was trained on a binary task—classifying whether and object was a plant or not. Only objects that were located closer than 3[m] from the sensing unit were classified. 0.035 s long echoes were used from both the right and left ear. These recordings were passed through three band pass filters, without the transmitted echo, (20-40kHz, 40-60kHz and 60-100kHz). Each echo was represented by 6 signals—3 filters x two ears. Next, a set of 21 acoustic features (Table 2) were extracted from each band-passed recording following T. Giannakopoulos [41]. Each echo was divided into seven windows equally spaced with an overlap of 40ms and the 21 features were extracted for each window generating a total of 147 dimensions per signal (21 features x 23 windows). The classifier was thus fed with 6 signals (483 dimensions each) and the decision of the majority of the six classifiers was used.

**Table 2:** Classification features.

Feture Name	Total
ZCR	23
Energy	46
Entropy	23
Spectral Centroid	23
Spectral Spread	23
Spectral Flux	23
Spectral Rolloff	23
Chrome Vector	299

The data was fed into a neural network with the following architecture:

Input layer—483 elements
First layer—105 elements with an RELU activation function
Dropout—0.5
Second Layer—50 elements with an RELU activation function
Third Layer—6 elements with an RELU activation function
Output Layer—1 element with a sigmoid activation function

We used Python’s TensorFlow to construct and train a three-layer neural-network (using the Keras directory).

The training sets included 788 plant examples and 628 non-plant examples collected on several sites on campus. We used the camera that was on the Robat to label the echoes.

Finally, to assess the statistical significance of our classification, we ran 100 permutations in which we assigned the training data randomly into the two classes (plants and non-plants), trained a classifier for each permutation and tested it on the same test-data.

We also tested several additional classification methods before choosing the neural-network. We tested a KNN (K nearest neighbors) classifier with five different distance measurements: Mahalanobis, Euclidean, Correlation, Minkowski and Canberra. We also tested two additional approaches for dimensionality reduction (before using the KNN) including PCA and LDA. In addition, we also tested a linear SVM classifier. For all classifiers, we used the same input features (see above).

The results were similar for most classifiers, but the neural network performed slightly better than the other (S8 Fig).

## Supporting information

S1 Fig The algorithm recognized and localized multiple reflectors within a single beam. Figure presents an echo train where three centers were localized. Spectrograms, correlation signals and the respective image (with centers) are depicted. Table shows the distances and azimuths of the three centers.
(TIF)

S2 Fig Acoustic mapping of the pteridophyte greenhouse. Colors and symbols are the same as in Fig 1. Red lines depict the actual borders of the greenhouse while blue lines show the borders estimated by the Robat. The black line shows the Robat’s movement in the greenhouse, a total of ca. 20m. The mean mapping error was 0.44 ± 0.25 [m] (mean + STD).
(TIF)

S3 Fig Localization accuracy estimated in the lab. Grey circles show real locations of a cylindrical object, while black crosses show estimated positions. Insert in bottom right shows an enlargement of the results when the object was at 0.8 m in front of the Robat.
(TIF)

S4 Fig The iterative obstacle Inflation algorithm. Each panel shows the result of another iteration of the algorithm,. The Robat used the map created after three iterations.
(TIF)

S5 Fig Mapping error estimation. (a) Real and estimated binary maps of the upper side of the greenhouse, showing the contour of the objects in white. (b) The same as in ‘a’ but with the two overlaid on-top of each other. (c) The same as in ‘b’, but after the 500 point interpolations with a 55 degree polynomial fit. (d) The error—distance between the real and the mapped contours.
(TIF)

S6 Fig Map reconstruction based on summing three echoes. The top three spectrograms represent three echoes recorded at the same location with different bearings. The bottom spectrogram represents the sum of the three echoes. The map on the bottom shows the result of using this mapping approach in the greenhouse (the same environment as in Fig 2a). Note that most of the errors are on one side, which was mostly composed of diffusive plant echoes.
(TIF)

S7 Fig Classification based decision making. Schematic and real images of the decision making task in which we drove the Robat into a dead end and let it decide which of the three sides is a plant through which it could pass.
(TIF)

S8 Fig The performance of different classification algorithms bars show correct classification of plants (blue) and non-plants (orange); BA—Balanced accuracy. (TIF)

## References

[1] Leonard JJ, Durrant-Whyte HF. Mobile robot localization by tracking geometric beacons. IEEE Transactions on Robotics and Automation. 1991;7(3):376–382. 10.1109/70.88147

[2] Tardos JD, Neira J, Newman PM, Leonard JJ. Robust Mapping and Localization in Indoor Environments Using Sonar Data. The International Journal of Robotics Research. 2002;21(4):311–330. 10.1177/027836402320556340

[3] Voigt CC, Frick WF, Holderied MW, Holland R, Kerth G, Mello MAR, et al Principles and Patterns of Bat Movements: From Aerodynamics to Ecology. The Quarterly Review of Biology. 2017;92(3):267–287. 10.1086/693847 29861509 PMC5983048

[4] Jensen ME. Echolocating bats can use acoustic landmarks for spatial orientation. Journal of Experimental Biology. 2005;208(23):4399–4410. 10.1242/jeb.01901 16339860

[5] Lindsay K, Roman K. Mobile Robot Sonar for Target Localization and Classification. The International Journal of Robotics Research. 1995;14(4):295–318. 10.1177/027836499501400401

[6] Vanderelst D, Steckel J, Boen A, Peremans H, Holderied M. Place recognition using batlike bio-sonar. eLife. 2016;1:1–15. 10.7554/eLife.14188 PMC4970868 27481189

[7] L K, R K. An optimal sonar array for target localization and classification. In: Proceedings of the 1994 IEEE International Conference on Robotics and Automation; 1994. p. 3130–3135 vol.4.

[8] Evers C, Moore AH, Naylor PA. Acoustic simultaneous localization and mapping (A-SLAM) of a moving microphone array and its surrounding speakers. ICASSP, IEEE International Conference on Acoustics, Speech and Signal Processing—Proceedings. 2016;2016-May:6Proceedings. 2016;2016-May:6–10.

[9] Vanderelst D, Holderied MW, Peremans H. Sensorimotor Model of Obstacle Avoidance in Echolocating Bats. PLoS Computational Biology. 2015;11(10):1–31. 10.1371/journal.pcbi.1004484 PMC4621039 26502063

[10] Ghose K. Steering by Hearing: A Bat’s Acoustic Gaze Is Linked to Its Flight Motor Output by a Delayed, Adaptive Linear Law. Journal of Neuroscience. 2006;26(6):1704–1710. 10.1523/JNEUROSCI.4315-05.2006 16467518 PMC3437256

[11] Yovel Y, Falk B, Moss CF, Ulanovsky N. Active control of acoustic field-of-view in a biosonar system. PLoS Biology. 2011;9(9). 10.1371/journal.pbio.1001150 21931535 PMC3172196

[12] Jakobsen L, Ratcliffe JM, Surlykke A. Convergent acoustic field of view in echolocating bats. Nature. 2013;493(7430):93–96. 10.1038/nature11664 23172147

[13] Reijniers J, Vanderelst D, Peremans H. Morphology-induced information transfer in bat sonar. Physical Review Letters. 2010;105(14):1–4. 10.1103/PhysRevLett.105.148701 21230873

[14] Genzel D, Wiegrebe L. Size does not matter: Size-invariant echo-acoustic object classification. Journal of Comparative Physiology A: Neuroethology, Sensory, Neural, and Behavioral Physiology. 2013;199(2):159–168. 10.1007/s00359-012-0777-3 23180047

[15] Simon R, Holderied MW, Koch CU, von Helversen O. Floral Acoustics: Conspicuous Echoes of a Dish-Shaped Leaf Attract Bat Pollinators. Science. 2011;333(6042):631–633. 10.1126/science.1204210 21798950

[16] Wotton JM, Simmons JA. Spectral cues and perception of the vertical position of targets by the big brown bat, Eptesicus fuscus. The Journal of the Acoustical Society of America. 2000;107(2):1034–1041. 10.1121/1.428283 10687712

[17] Yovel Y, Stilz P, Franz MO, Boonman A, Schnitzler HU. What a plant sounds like: The statistics of vegetation echoes as received by echolocating bats. PLoS Computational Biology. 2009;5(7). 10.1371/journal.pcbi.1000429 PMC2699101 19578430

[18] Yovel Y, Franz MO, Stilz P, Schnitzler HU. Plant classification from bat-like echolocation signals. PLoS Computational Biology. 2008;4(3). 10.1371/journal.pcbi.1000032 18369425 PMC2267002

[19] Yamada Y, Ito K, Oka A, Tateiwa S, Ohta T, Kobayashi R, et al Obstacle-Avoidance Navigation by an Autonomous Vehicle Inspired by a Bat Biosonar Strategy In: Biomimetic and Biohybrid Systems. Cham: Springer International Publishing; 2015 p. 135–144.

[20] Peremans H, Mey FD, Schillebeeckx F. Man-made versus biological in-air sonar systems Frontiers in Sensing: From Biology to Engineering. 2012;9783211997:195–207. 10.1007/978-3-211-99749-9_13

[21] Steckel J, Peremans H. BatSLAM: Simultaneous localization and mapping using biomimetic sonar. PloS One. 2013;8(1):e54076 10.1371/journal.pone.0054076 23365647 PMC3554696

[22] Brooks RA. A Robust Layered Control System For A Mobile Robot. IEEE Journal on Robotics and Automation. 1986;2(1):14–23. 10.1109/JRA.1986.1087032

[23] Surlykke A, Ghose K, Moss CF. Acoustic scanning of natural scenes by echolocation in the big brown bat, Eptesicus fuscus. Journal of Experimental Biology. 2009;212(7):1011–1020. 10.1242/jeb.024620 19282498 PMC2726860

[24] Amichai E, Blumrosen G, Yovel Y. Calling louder and longer: how bats use biosonar under severe acoustic interference from other bats. Proceedings of the Royal Society B: Biological Sciences. 2015;282(1821):20152064 10.1098/rspb.2015.2064 26702045 PMC4707756

[25] HU S, CF M, A D. From spatial orientation to food acquisition in echolocating bats. Trends in Ecology and Evolution. 2003;18(8):386–394. 10.1016/S0169-5347(03)00185-X

[26] Nihoul JCJ. Echolocation in Bats and Dolphins. Journal of Marine Systems. 2004; 10.1016/j.jmarsys.2004.01.009

[27] Daniell H. Flying big brown bats emit a beam with two lobes in the vertical plane. J Acoust Soc Am 2007. 2012;76(October 2009):211–220. 10.1121/1.2799491 PMC3397164 18247779

[28] Jakobsen L, Brinkl⌀v S, Surlykke A. Intensity and directionality of bat echolocation signals. Frontiers in Physiology. 2013;4:89 10.3389/fphys.2013.00089 23630501 PMC3635024

[29] Kounitsky P, Rydell J, Amichai E, Boonman A, Eitan O, Weiss AJ, et al Bats adjust their mouth gape to zoom their biosonar field of view. Proceedings of the National Academy of Sciences. 2015;112(21):6724–6729. 10.1073/pnas.1422843112 PMC4450403 25941395

[30] Yufka A, Parlaktuna O. Performance Comparison of BUG Algorithms for Mobile Robots. Performance Comparison of BUG Algorithms for Mobile Robots. 2009;p. 61–65.

[31] Simmons JA. Perception of echo phase information in bat sonar; 1979. 10.1126/science.451543 451543

[32] Simmons JA. The resolution of target range by echolocating bats. The Journal of the Acoustical Society of America. 1973;54(1):157–173. 10.1121/1.1913559 4738624

[33] M O Franz PSYY. Complex echo classification by echo-locating bats: a review. Journal of Comparative Physiology. 2011;197:475–490. 10.1007/s00359-010-0584-7 20848111

[34] Grinnell A. Hearing in Bats: An Overview; 1995.

[35] Heffner RS, Koay G, Heffner HE. Sound localization in common vampire bats: Acuity and use of the binaural time cue by a small mammal. The Journal of the Acoustical Society of America. 2015;137(1):42–52. 10.1121/1.4904529 25618037 PMC4304952

[36] Heffner RS, Koay G, Heffner HE. Sound localization in a new-world frugivorous bat, Artibeus jamaicensis: Acuity, use of binaural cues, and relationship to vision. The Journal of the Acoustical Society of America. 2001;109:412–421. 10.1121/1.1329620 11206172

[37] Bar NS, Skogestad S, Marcal JM, Ulanovsky N, Yovel Y. A Sensory-Motor Control Model of Animal Flight Explains Why Bats Fly Differently in Light Versus Dark. PLoS Biology. 2015;13(1):1–18. 10.1371/journal.pbio.1002046 PMC4309566 25629809

[38] Sigman M, Dehaene S. Brain Mechanisms of Serial and Parallel Processing during Dual-Task Performance. Journal of Neuroscience. 2008;28(30):7585–7598. 10.1523/JNEUROSCI.0948-08.2008 18650336 PMC6670853

[39] Lumelsky V, Skewis T. Incorporating Range Sensing in the Robot Navigation Function. IEEE Transactions on Systems, Man and Cybernetics. 1990;20(5):1058–1069. 10.1109/21.59969

[40] Kümmerle R, Steder B, Dornhege C, Ruhnke M, Grisetti G, Stachniss C, et al On measuring the accuracy of SLAM algorithms. Autonomous Robots. 2009;27(4):387–407. 10.1007/s10514-009-9155-6

[41] Giannakopoulos T. pyAudioAnalysis: An Open-Source Python Library for Audio Signal Analysis. PLOS ONE. 2015 12;10(12):1–17. 10.1371/journal.pone.0144610 PMC4676707 26656189

