# Supplementary figures and images for: A fully autonomous terrestrial bat-like acoustic robot
Source: PLoS Comput Biol. 2018 Sep 6;14(9):e1006406. doi: 10.1371/journal.pcbi.1006406 (PMC6126821; doi:10.1371/journal.pcbi.1006406)

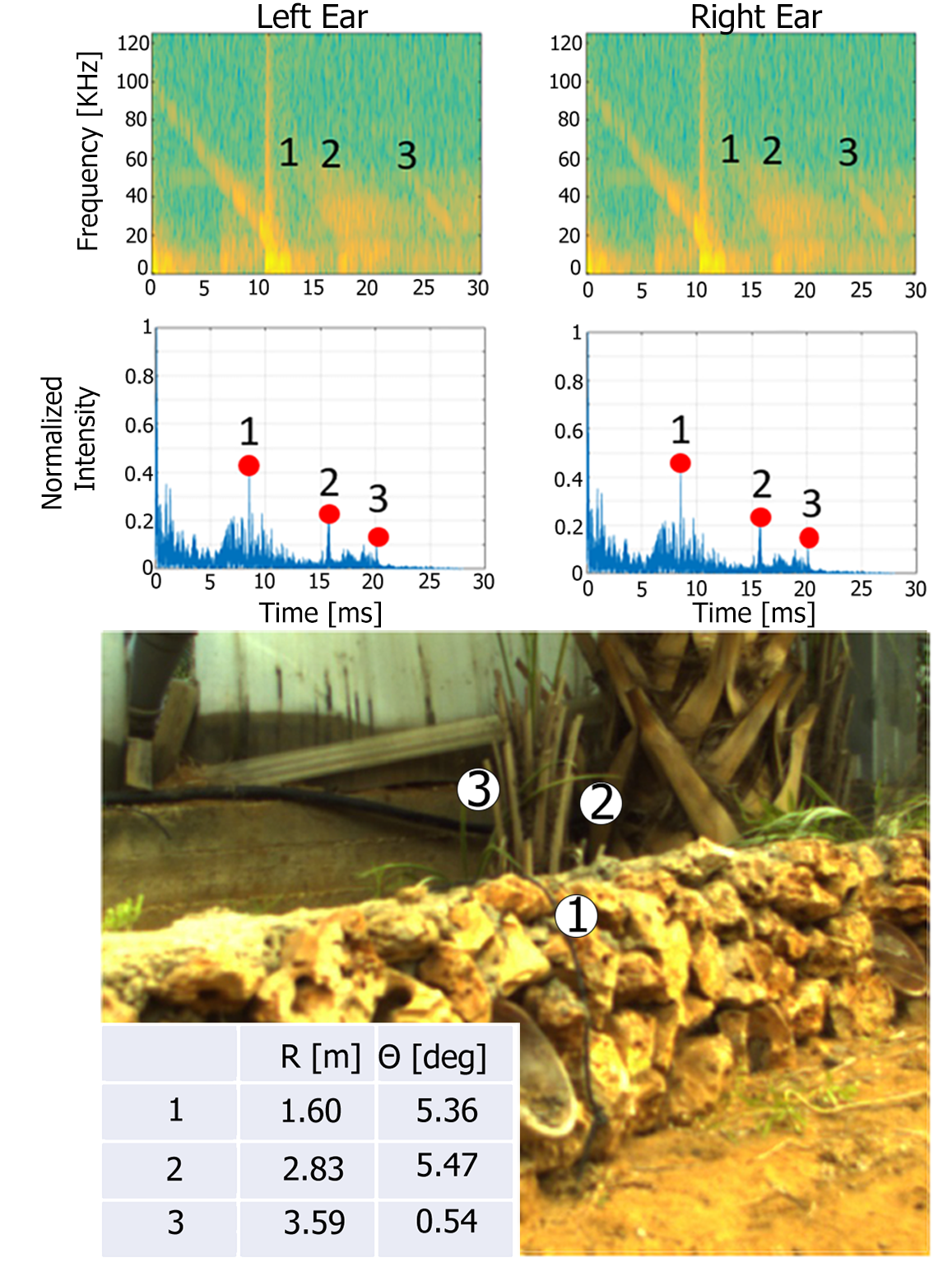

Supplement: S1 Fig — Figure presents an echo train where three centers were localized. Spectrograms, correlation signals and the respective image (with centers) are depicted. Table shows the distances and azimuths of the three centers. (TIF) [file pcbi.1006406.s001.tif]

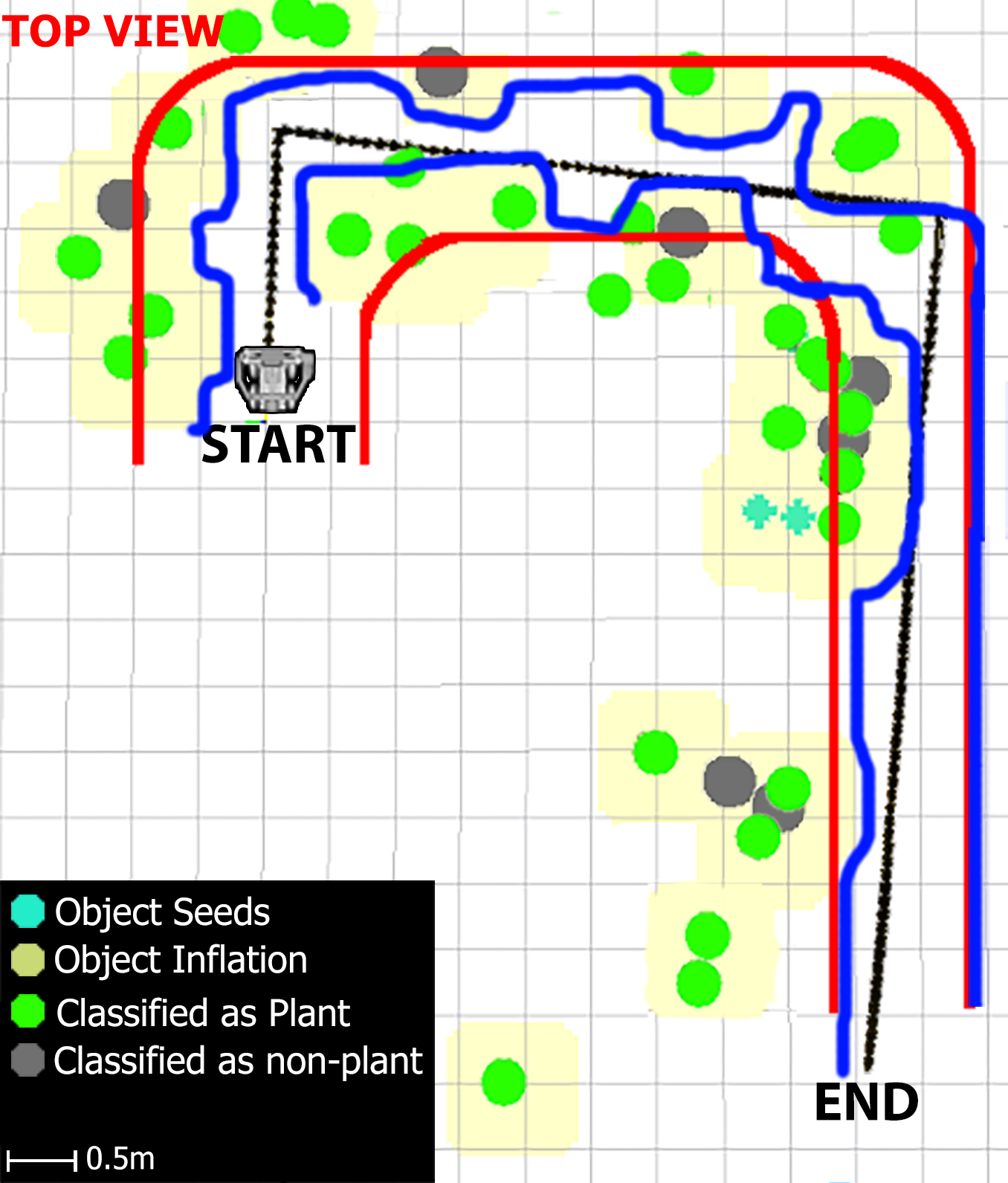

Supplement: S2 Fig — Colors and symbols are the same as in Fig 1. Red lines depict the actual borders of the greenhouse while blue lines show the borders estimated by the Robat. The black line shows the Robat’s movement in the greenhouse, a total of ca. 20m. The mean mapping error was 0.44 ± 0.25 [m] (mean + STD). (TIF) [file pcbi.1006406.s002.tif]

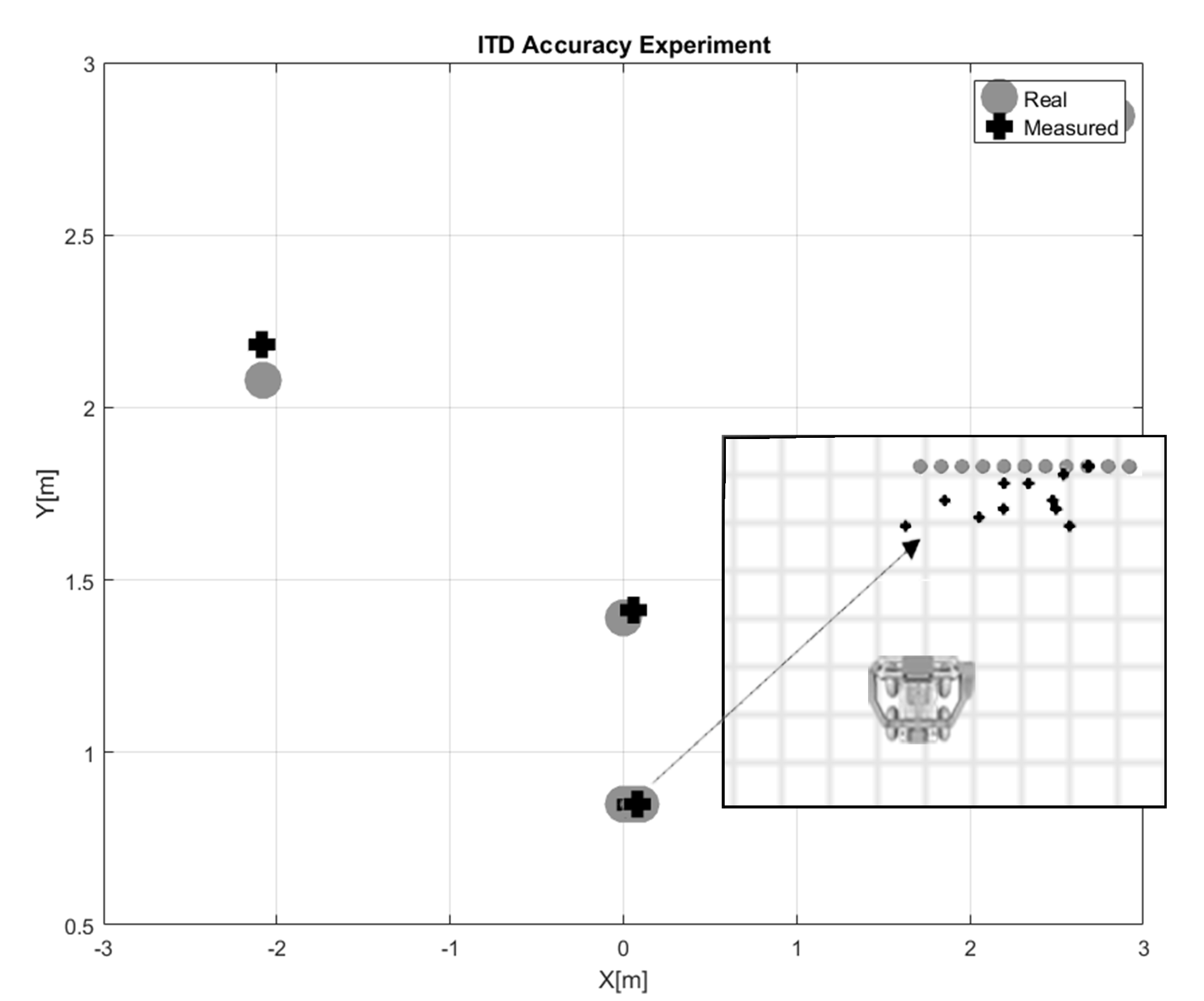

Supplement: S3 Fig — Grey circles show real locations of a cylindrical object, while black crosses show estimated positions. Insert in bottom right shows an enlargement of the results when the object was at 0.8 m in front of the Robat. (TIF) [file pcbi.1006406.s003.tif]

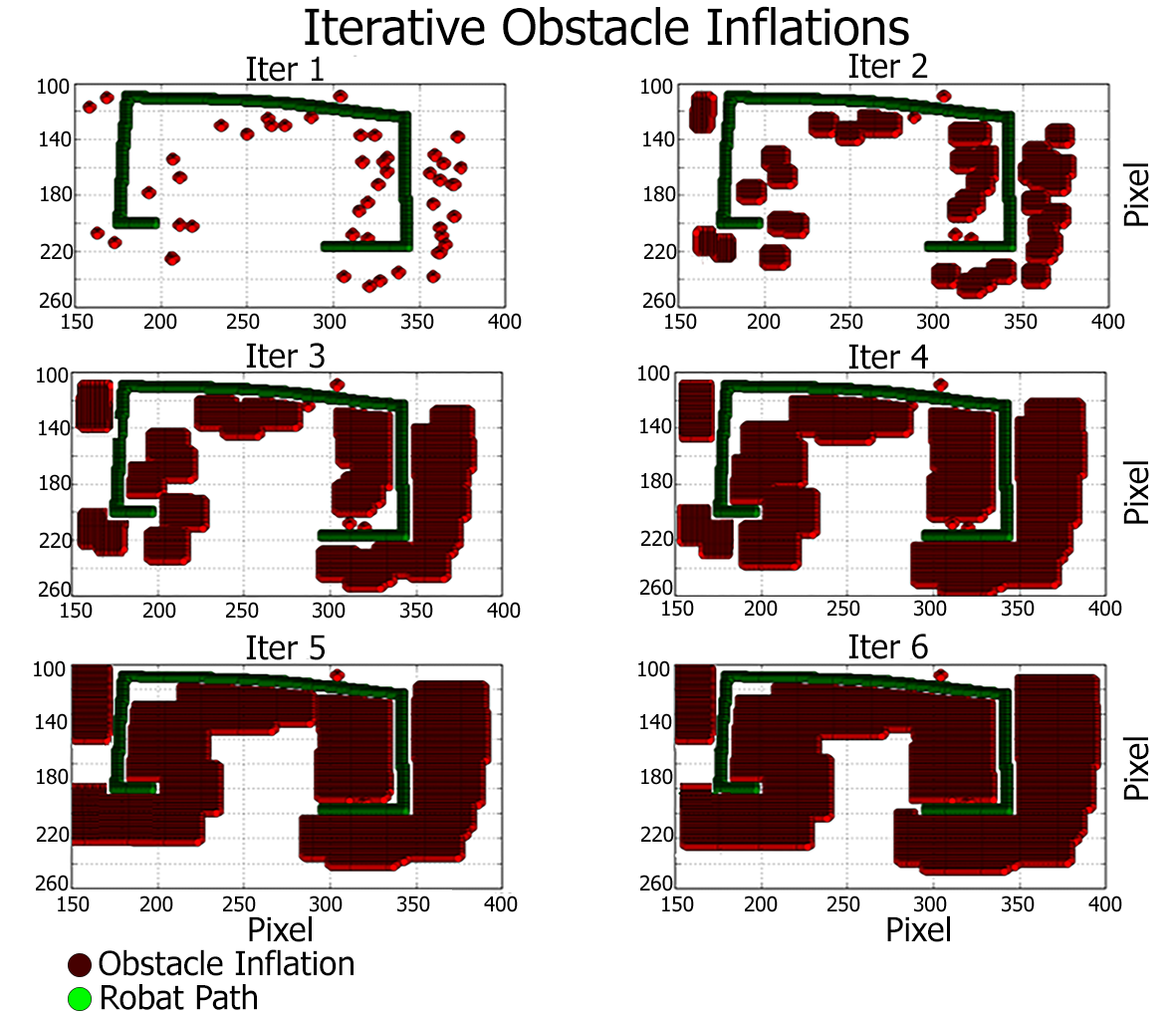

Supplement: S4 Fig — Each panel shows the result of another iteration of the algorithm,. The Robat used the map created after three iterations. (TIF) [file pcbi.1006406.s004.tif]

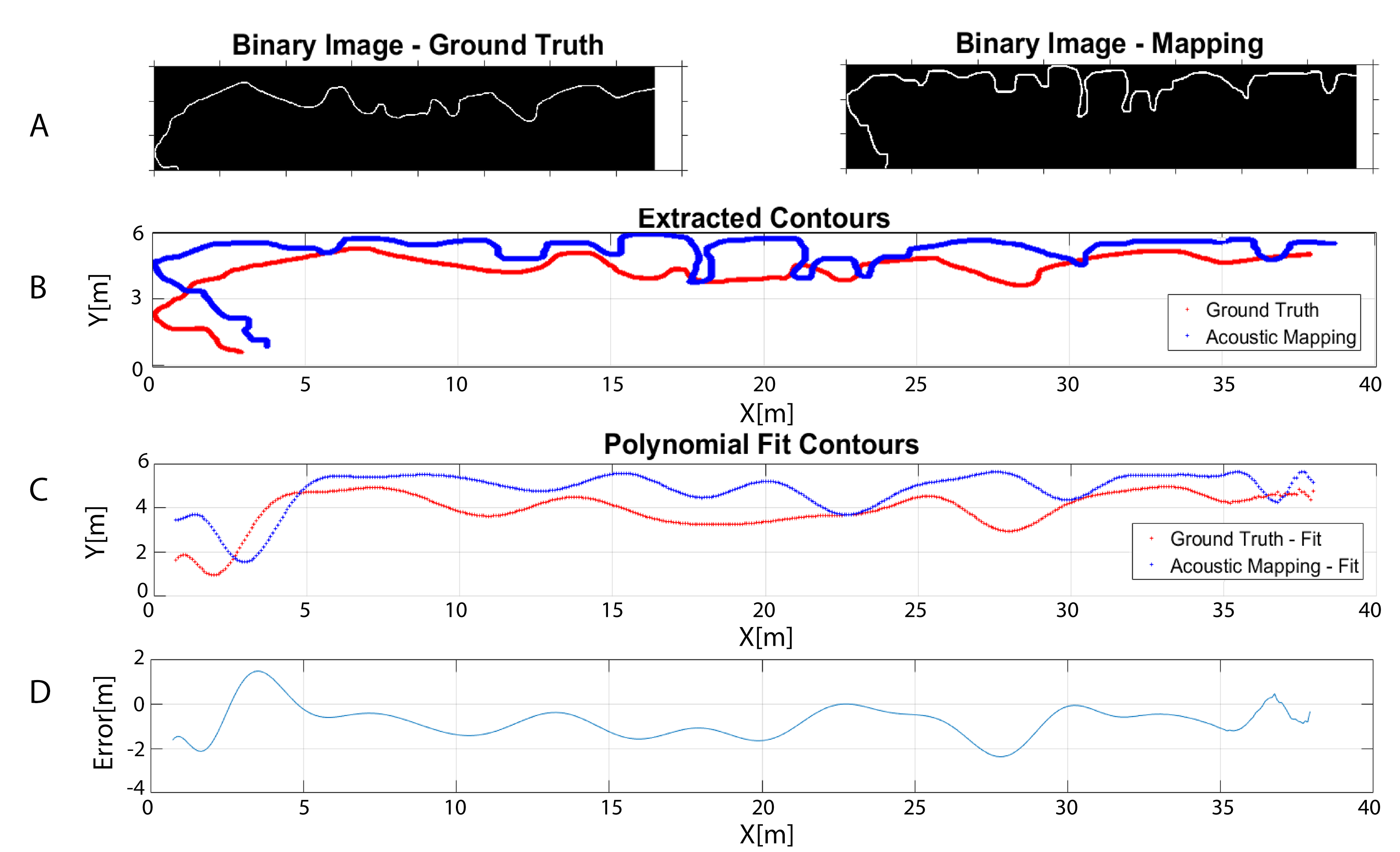

Supplement: S5 Fig — (a) Real and estimated binary maps of the upper side of the greenhouse, showing the contour of the objects in white. (b) The same as in ‘a’ but with the two overlaid on-top of each other. (c) The same as in ‘b’, but after the 500 point interpolations with a 55 degree polynomial fit. (d) The error—distance between the real and the mapped contours. (TIF) [file pcbi.1006406.s005.tif]

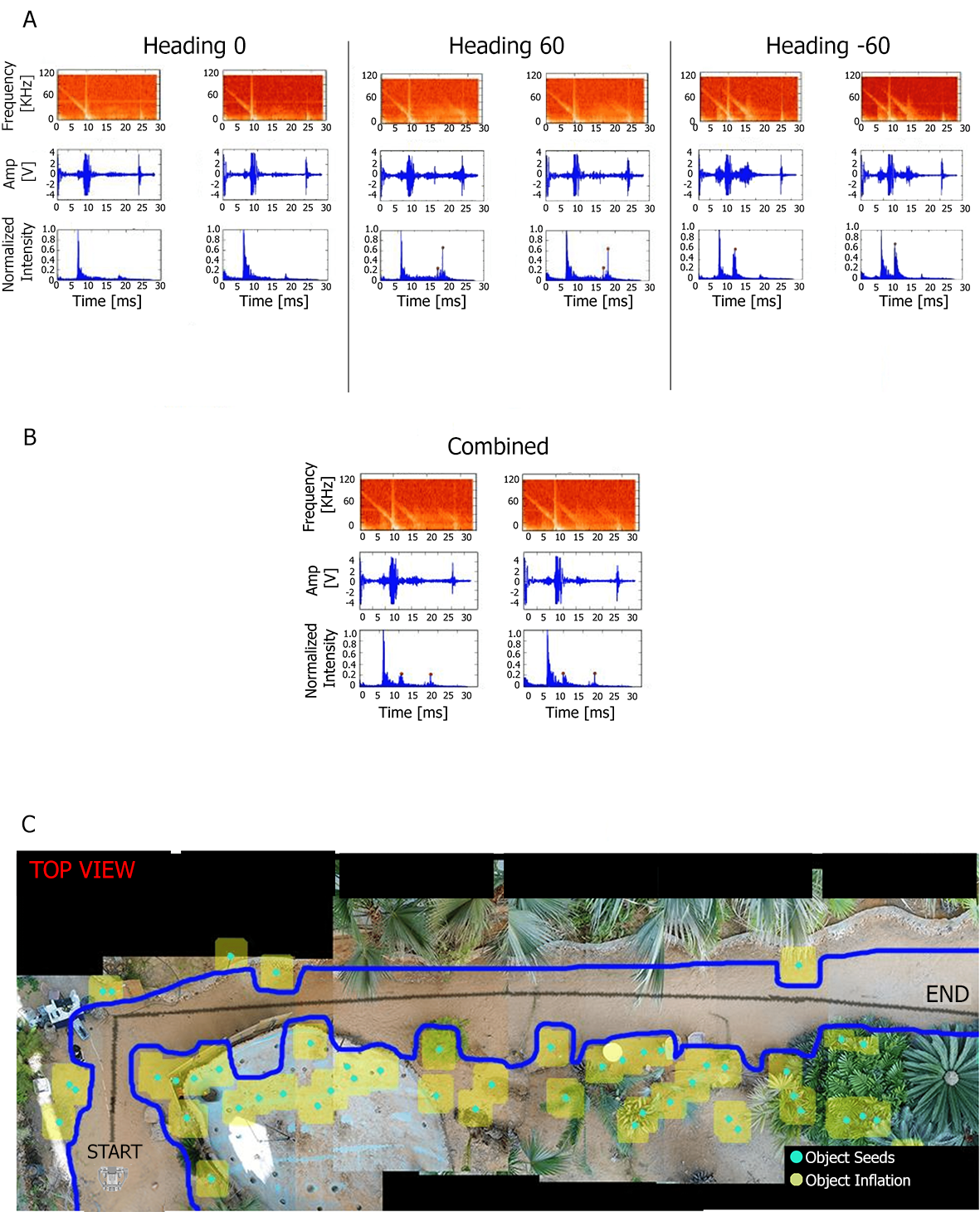

Supplement: S6 Fig — The top three spectrograms represent three echoes recorded at the same location with different bearings. The bottom spectrogram represents the sum of the three echoes. The map on the bottom shows the result of using this mapping approach in the greenhouse (the same environment as in Fig 2a). Note that most of the errors are on one side, which was mostly composed of diffusive plant echoes. (TIF) [file pcbi.1006406.s006.tif]

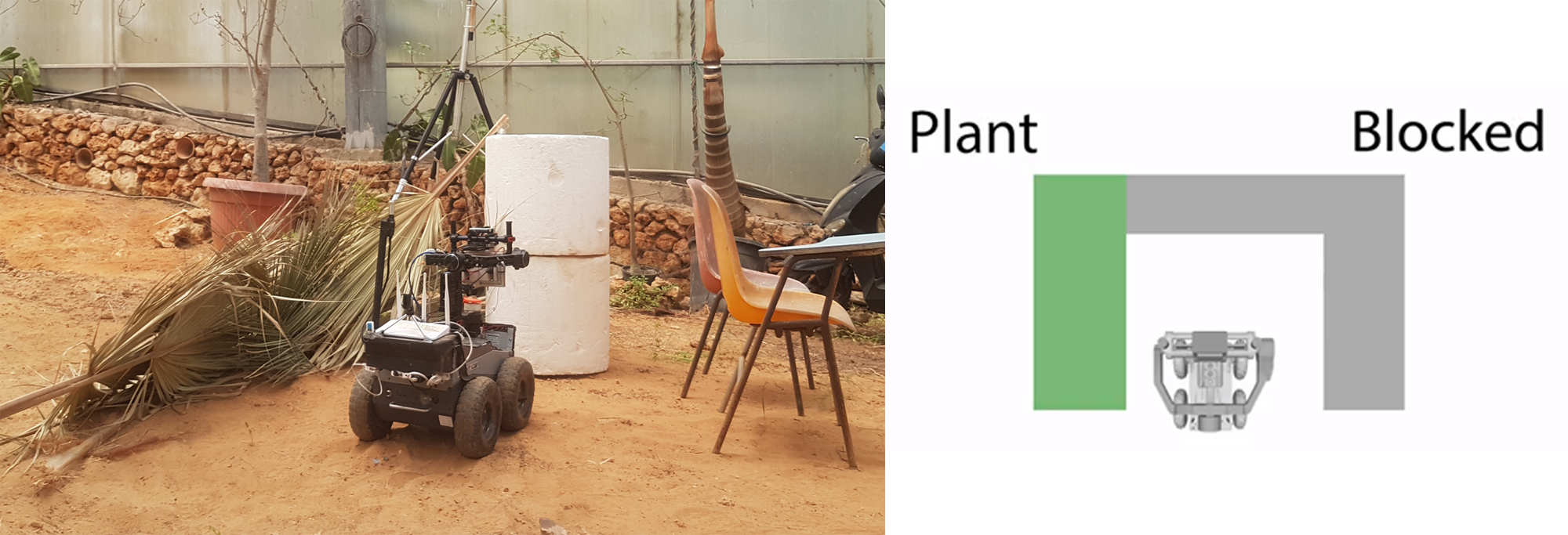

Supplement: S7 Fig — Schematic and real images of the decision making task in which we drove the Robat into a dead end and let it decide which of the three sides is a plant through which it could pass. (TIF) [file pcbi.1006406.s007.tif]

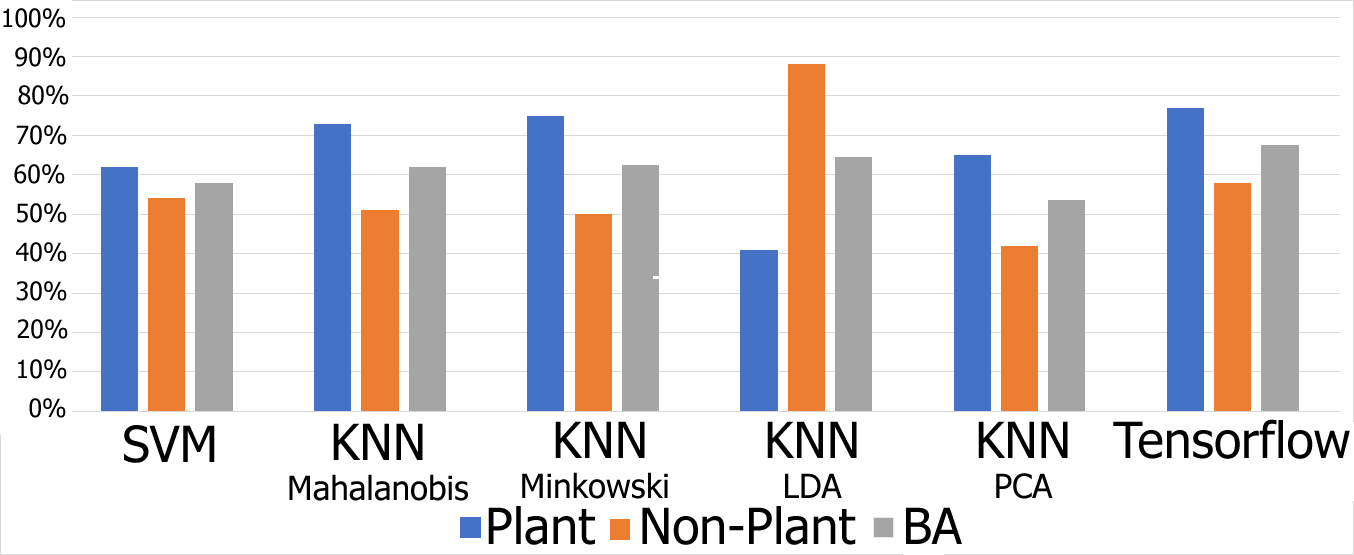

Supplement: S8 Fig — (TIF) [file pcbi.1006406.s008.tif]
